# Smarter ways to predict rabbit body weight across multiple breeds

**DOI:** 10.5455/javar.2025.l964

**Published:** 2025-09-23

**Authors:** Bram Brahmantiyo, Henny Nuraini, Amelia Kamila Islami, Rini Herlina Mulyono, Galih Ari Wirawan Siregar, Ferdy Saputra, Mohammad Ikhsan Shiddieqy, Nurul Azizah, Cecep Hidayat, Wawan Sulistiono

**Affiliations:** 1 Research Center for Animal Husbandry, National Research and Innovation Agency of the Republic of Indonesia, Cibinong, Indonesia; 2 Department of Animal Production and Technology, Faculty of Animal Science, IPB University, Bogor, Indonesia; 3 Animal Production Program, Faculty of Agriculture, Medan, Indonesia; 4 Animal Production Systems Group, Wageningen University & Research, Wageningen, The Netherlands

**Keywords:** Rabbit, Morphometrics, Linear models, Regression analysis, Random forest

## Abstract

**Objective:**

Morphometric measurement is essential in the determination of breeding program zones that need to be improved.

**Materials and Methods:**

This research aims to compare the precision of morphometric measurement to linear models, such as regression analysis and machine learning methods, such as Random Forest (RF), to improve the precision of live weight estimation in animal breeding programs. A total of 228 rabbits were used in the current study, and they comprised the following breeds:39 Satin, 40 Rex, 40 New Zealand White, 29 Hyla, 40 Hycole, and 40 Reza were utilized for the study. Each rabbit was measured on body weight, head (width and length), chest circumference, body length, and hip width. Stepwise regression and linear regression analyses were conducted using the lm function in R version 4.4.1. For the RF algorithm, the caret and randomForest packages were utilized to build and evaluate the model.

**Results:**

In this study, linear regression [R-squared value of 0.82 and an Root Mean Squared error (RMSE) of 300.16] outperformed RF (R-squared value of 0.8 and an RMSE of 326.37) in predicting rabbit body weight based on morphometric measurements. The results showed that chest circumference and body length were the most influential predictors, with the largest coefficients and highest significance levels, and the IncNodePurity illustration showed head length (IncNodePurity: 19388974) emerged as an important factor in predicting body weight.

**Conclusion:**

The Linear regression model showed superior results compared to the RF model in predicting rabbit body weight based on morphometric measurements.

## Introduction

Rabbits can be utilized both for meat production and as pets. Their small size, docile temperament, and rapid reproductive rate make them well-suited for these roles [1]. For meat production, rabbits possess a number of unique benefits, such as a high rate of reproduction [2,3]. In relation to other animals, rabbits require little space and resources to raise, and thus, they consume less in terms of the environment.

Rabbit meat is also becoming increasingly acceptable in Indonesia due to the increasing demand for alternative protein sources [4,5], and rabbit meat has the immense scope to provide sustainable income to the small farmers [6]. Rabbit is a very good source of nutrition since it consists of high protein content, extremely low fat content, and a desirable percentage of unsaturated fatty acids. In addition, rabbit meat contains virtually no cholesterol and consists mainly of calcium and phosphorus, thus being a healthy choice of food [7].

Morphometric measurements play an important role in the classification and differentiation of various breeds of animals [8–10]. This study can prove to be helpful in the knowledge about how different populations have evolved and how one can maximize the changes that can be enhanced in the breeding programs. Furthermore, morphometrics is a reliable predictor of carcass weight and body weight of animals [11,12]. Such an approach is particularly appropriate in the livestock production sector, where meat yield and performance of growth may be tested non-invasively and to an economically reasonable degree.

Moreover, linear models and machine learning software can be used to significantly improve prediction accuracy in the field of animal science, such as the estimation of live weight [13,14]. Linear models such as regression analysis offer a simple and interpretable method for determining the relationship between morphometric measurements and factors such as body weight or carcass yield. Conversely, machine learning algorithms like Random Forest (RF) have superior attributes to identify a non-linear pattern in data. Thus, the purpose of this research is to compare and evaluate the linear regression and RF algorithm to determine how efficient they are in forecasting the body weight from the measurements of the body size. Through these two different modeling techniques, the research aims to determine which of the two methods is more accurate and reliable in predicting body weight from morphometric measurements.

## Materials and Methods

### Ethical approval

This study was carried out at the Indonesian Research Institute for Animal Production in Ciawi, Indonesia. Approval for this study was sought from the Institutional Animal Care and Use Committee of the Indonesian Agency for Agricultural Research and Development with the approval number Balitbangtan/Balitnak/Rd/01/2021.

### Animals

A total of 228 6-month-old rabbits were used in this study, comprising 39 Satin (20 male and 19 female), 40 Rex (20 male and 20 female), 40 New Zealand White (20 male and 20 female), 29 Hyla (9 male and 20 female), 40 Hycole (20 male and 20 female), and 40 Reza (16 male and 24 female) breeds. The kits were weaned at 5 weeks. The diet used in this study contained 18% crude protein, 2,500 kcal/kg metabolic energy, and 14% crude fiber. The diet was made in the form of a pellet. The pellet was delivered in the morning and evening, with drinking water supplied ad libitum.

### Statistical analysis

Each rabbit individual was measured on body weight, head (width and length), chest circumference, body length, and hip width. Stepwise regression and linear regression analyses were conducted using the lm function in R version 4.4.1. For the RF algorithm, the caret and randomForest packages were utilized to build and evaluate the model. Data visualization and plotting were performed using the ggplot2 package, ensuring clear and informative graphical representations of the results.

## Results and Discussion

The model of stepwise regression shows a significant result of the variance in body weight, as indicated by the highly significant predictor values (head length, chest circumference, and body length) (Tables 1 and 2). Chest circumference and body length are the most influential predictors, with the largest coefficients and the highest significance levels. This suggests that these variables can accurately predict body weight.

**Table 1.** Dataset for predicting body weight using morphometric data.

Breed	Sex	Body weight (gm)	Head length (cm)	Head width (cm)	Chest circumference (cm)	Body length (cm)	Hip width (cm)
Satin	Male (*n* = 20)	2,066.25 ± 251.25	11.24 ± 0.88	4.59 ± 0.29	29.22 ± 2.59	31.53 ± 1.68	8.90 ± 1.17
	Female (*n* = 19)	2,568.16 ± 404.66	13.25 ± 1.11	4.41 ± 0.66	31.69 ± 2.81	33.21 ± 1.14	8.47 ± 0.83
Rex	Male (*n* = 20)	2,579.25 ± 421.85	12.87 ± 1.39	5.12 ± 0.49	31.61 ± 1.93	33.48 ± 1.04	8.84 ± 0.56
	Female (m = 20)	1,829.50 ± 269.10	11.18 ± 1.43	4.14 ± 0.53	27.47 ± 2.58	30.39 ± 1.69	8.52 ± 1.01
New Zealand White	Male (*n* = 20)	3,434 ± 566.54	13.75 ± 0.97	5.22 ± 0.68	32.45 ± 2.26	37.33 ± 3.13	8.93 ± 0.66
	Female (*n* = 20)	3,024.50 ± 402.04	13.93 ± 0.82	4.80 ± 0.34	31.54 ± 1.29	35.76 ± 1.91	8.63 ± 0.59
Hyla	Male (*n* = 9)	3,397.78 ± 455.96	14.47 ± 0.65	5.08 ± 0.35	34.13 ± 2.14	34.93 ± 1.23	8.08 ± 0.95
	Female (*n *= 20)	3,492.25 ± 701.55	13.76 ± 1.89	4.90 ± 0.34	33.96 ± 2.35	36.31 ± 2.39	9.13 ± 1.05
Hycole	Male (*n* = 20)	3,377.50 ± 523.49	14.25 ± 1.18	5.01 ± 0.34	34.16 ± 2.49	37.73 ± 1.60	9.22 ± 0.69
	Female (*n *= 20)	3,379.50 ± 601.14	13.73 ± 2.36	4.69 ± 0.34	33.56 ± 2.09	37.37 ± 1.53	8.97 ± 0.71
Reza	Male (*n* = 16)	2,392.19 ± 318.82	12.91 ± 1.06	4.81 ± 0.24	30.26 ± 1.28	33.92 ± 1.18	8.14 ± 4.94
	Female (*n* = 24)	2,271.46 ± 324.12	13.10 ± 0.51	4.43 ± 0.23	30.17 ± 1.47	33.72 ± 1.82	7.92 ± 0.47

The numbers in the table are (mean ± SE)

**Table 2.** Stepwise regression results for body weight prediction.

Variable	Estimate	Std. Error	*t*-value	*p*-value	Significance
Intercept	−5,797.995	259.508	−22.342	< 2e–16	***
Head length	60.519	15.174	3.988	9.04e–05	***
Head width	73.511	41.386	1.776	0.0771	.
Chest circumference	116.409	9.966	11.680	< 2e-16	***
Body length	95.233	9.403	10.128	< 2e-16	***
Hip width	54.446	25.505	2.135	0.0339	*

*:*p* < 0.05, **:*p* < 0.01, ***:*p* < 0.001

Head width and hip width are less likely to contribute to the model, indicating that they provide additional explanatory power. The intercept suggests that the model predicts a negative body weight when all predictors are zero, which is biologically implausible. This indicates that the relationship between the predictors and body weight is not linear across the entire range of values, or that the model may not extrapolate well outside the observed data range.

The findings are consistent with previous studies on other species, such as Belgian Blue cattle, where Chest Circumference and Body Length were identified as the most important predictors of body weight [15]. This supports the belief that such morphometric characteristics are strong universal predictors of body mass across various animal species. The same outcome is attained in more recent research on goats and sheep, whose chest girth and body length have been significantly correlated with body weight as predictors in models of body weight prediction [16,17].

**Figure 1.**
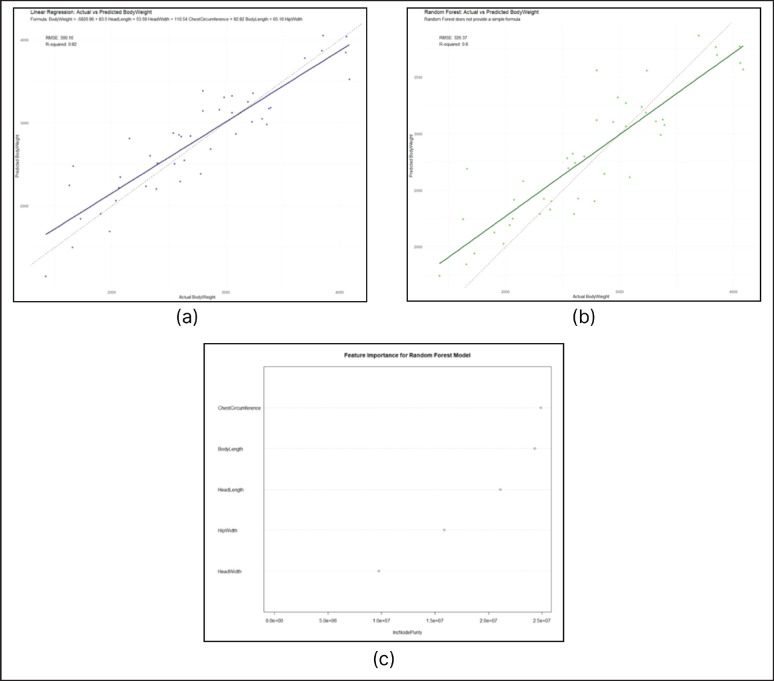
(a) Linear regression for predicting body weight using morphometric data in Indonesian rabbits. (b) Random forest for predicting body weight using morphometric data in Indonesian rabbits. (c) Variable of importance in a random forest.

The linear regression model that was applied to forecast the body weight of the rabbits based on predictors like chest circumference, body length, head length, and hip width had an R-squared of 0.82 and an Root Mean Squared error (RMSE) of 300.16 (Fig. 1a). These findings can be useful to explain the performance of the model and relations between the predictors and body weight. An R-squared value of 0.82 indicates that the model accounts for a large part of the underlying relationship between these morphometric measurements and body weight.

The RF model that was used to predict the body weight of the rabbits based on predictors, which included chest circumference, body length, head length, and hip width, had an R-squared of 0.8 and an RMSE of 326.37. (Fig. 1b). Linear regression was better than RF for rabbit body weight prediction based on morphometric measurements in this study. The finding is consistent with Ruchay et al. [18], who found that linear regression gave improved body weight predictions in Hereford cows as compared to support vector machines and RF.

In addition, the development of machine learning algorithms has also confirmed the significance of these morphometric traits. For instance, a study on pigs demonstrated the StackingRegressor model for predicting body weight, achieving high accuracy and robustness [19]. Similarly, poultry research highlighted the utility of combining traditional morphometric measurements with machine learning algorithms to improve weight prediction accuracy, particularly in large-scale farming operations [20]. The findings from this comparison will contribute to the development of more effective tools for weight prediction, supporting improved livestock management and breeding practices. However, simpler models may be more effective when the relationships between predictors and body weight are predominantly linear.

Figure 1c illustrates IncNodePurity (Increase in Node Purity), a metric used in RF models to assess the importance of predictor variables. In the context of this study, IncNodePurity for chest circumference (IncNodePurity: 27698337) and body length (IncNodePurity: 25253933) are most influential in predicting body weight. Interestingly, head length (IncNodePurity: 19388974) emerges as an important factor in predicting body weight in this study. This finding is unexpected, as head length is not commonly identified as a significant predictor in body weight estimation models. For instance, Abbas et al. [21] found no significant effect of head length in predicting body weight in sheep. The prominence of head length in this study could be attributed to unique breed characteristics or specific morphometric relationships in the studied population.

Machine learning became an essential tool in modern livestock management, offering innovative solutions for monitoring animal health, predicting body weight, optimizing breeding programs, and enhancing overall farm efficiency. Algorithms such as RF [22,23], Support Vector Machine [24,25], Artificial neural networks [26,27], Convolutional Neural Network [28,29], and Gradient Boosting [30,31] were successfully applied to analyze morphometric data, classify animal behavior, predict growth performance, predict electricity consumption on dairy farms, and predict milk yield.

Moreover, RF had a higher accuracy compared to Gradient Boosting and Support Vector Machine in determining active and inactive activities in growing rabbits [32]. In addition, the use of machine learning in rabbit farming can be used for image recognition of fecal shape to monitor rabbit health [33]. The integration of machine learning with the Internet of Things (IoT) is expected to enhance rabbit farm management by enabling real-time monitoring, optimizing growth performance, and improving breeding selection programs. IoT devices, such as smart sensors and cameras, can continuously collect data on environmental conditions, feeding patterns, and rabbit behavior, while machine learning algorithms analyze this data to detect health issues, predict growth rates, and recommend optimal breeding pairs. This advanced approach can lead to more efficient resource utilization, reduced production costs, and improved overall farm productivity.

## Conclusion

In conclusion, chest circumference, body length, and head length are reliable predictors of body weight for rabbits. The linear regression model showed superior results compared to the RF model in predicting rabbit body weight based on morphometric measurements.

## List of abbreviations

RMSE: Root Mean Squared Error; RF: Random Forest.
